# The Late Epidemic

**Published:** 1898-02

**Authors:** 


The Late Epidemic.—The extra expense to the State of guarding against invasion of yellow fever last fall, an expense not foreseen and not provided for, was, in round numbers, $4500. That covers the entire expense of the inspection station and service two months at Texarkana, Atlanta, Waskom, Logansport, Possum Gap and Sabine River Crossing, the State Health Officer's traveling expenses,—everything. The appropriation for the Quarantine Department, for all purposes, is $33,000 a year. Notwithstanding the extra expense above stated, the deficiency will amount to only $3500, or an amount sufficient to pay the regular expenses for the months of January and February. The fiscal year begins March 1st. The appropriation for the year 1898, $33,000, cannot be used to meet this deficiency, and the quarantine officers and empleyes will have to wait for their pay until the next Legislature makes an appropriation for the purpose of paying it. If the law permitted the State Health Officer to trench upon the appropriation for 1898; if the appropriations were made for two years, $66,000, instead of $33,000 for each year, it is believed that by skillful management and strict economy the deficiency could be wiped out before the end of 1898. But the appropriation for one year cannot be used for expenses for another year.

It is to be hoped that the Legislature may be made now to see the necessity of putting a contingent fund, say of $5000, at the disposal of the State Health Officer, with which to meet such unexpected occurrences. It is embarrassing, and it is unjust, that the officers of the department should have to wait for their heard-earned wages. If not needed, it would not be used, so—what's the odds?

---

Rush Medical College has entered into an arrangement with the University of Chicago whereby the trustees of the University will have a general supervision over the educational policy of that college, the agreement to take effect as soon as certain details or preliminaries are adjusted—the payment of a debt of $71,000 being one of them. Another condition is that the Board of Trustees of the Rush shall be reorganized. The doctors on the board who are also professors are to be replaced by business men who have no pecuniary interest in the success or failure of the college. It is hoped to effect the change June 1, 1898.

Prof. Harper, of the University of Chicago, in an address recently, made the statement that every time Rush College raised its standard, imposed additional requirements, its classes increased—a most remarkable, a unique experience. Why not continue to raise it, even to the impossible? As a matter of fact, it is announced that after 1902 no one will be permitted to matriculate except those who have finished both freshman and sophomore courses at some college.

When all the medical colleges can do this we may expect a higher standard of medical education and better average doctors. But we had as well look for the millenium. We wish Rush may be able to fill its benches every year after 1902, but we doubt it, so long as there are reputable colleges that have a lower standard.

---

"JUST AS I EXPECTED," will say a lot of fellers, when they read this. Now, hold on; don't say it before you know what it is about.

Dr. Willis King wrote a book.

Dr. Frank Lydston wrote a book.

Dr. Weir Mitchell wrote a book.

Dr. Ian MacLaren wrote a book.

A whole lot of fellers wrote a book.

Now, why shouldn't *I* write a book? Somebody said—"Oh, if my enemy would write a book"—(I forgot what he said he'd do if he did.) Well, I ain't anybody's enemy in particular; but I've written a book all the same. It will be published pretty soon, now; just as soon as I can get somebody to publish it, and (it's already written) it will be called

"RECOLLECTIONS OF A REBEL SURGEON;"

Or, "In the Doctor's Sappy Days." A series of short sketches, personal reminiscenses—mostly humorous—of life in camp, field and hospital "endurin' of the war"—by F. E. Daniel, M. D., editor TEXAS MEDICAL JOURNAL, late surgeon C. S. A., etc., and want every fellow who owns, controls or runs a medical journal on shares—and who has a kindly feeling for yours truly and any kind of regard for Betty and the baby, to announce the important fact;—say it out loud; in a way calculated to make the book sell; (don't say it is a "sell," but help make it sell, see?) It's real good.

